# Reconstitution of the Mevalonate Pathway for Improvement of Isoprenoid Production and Industrial Applicability in *Escherichia coli*

**DOI:** 10.4014/jmb.2408.08053

**Published:** 2024-10-11

**Authors:** Min-Kyoung Kang, Minh Phuong Nguyen, Sang-Hwal Yoon, Keerthi B. Jayasundera, Jong-Wook Son, Chonglong Wang, Moonhyuk Kwon, Seon-Won Kim

**Affiliations:** 1 Anti-aging Bio Cell factory Regional Leading Research Center, Gyeongsang National University, Jinju 52828, Republic of Korea; 2 Division of Applied Life Science (BK21 Four), Gyeongsang National University, Jinju 52828, Republic of Korea; 3 School of Biology and Basic Medical Sciences, Soochow University, Suzhou 215123, People's Republic of China; 4 Research Institute of Molecular Alchemy (RIMA), Gyeongsang National University, Jinju 52828, Republic of Korea; 5 Plant Molecular Biology & Biotechnology Research Center, Gyeongsang National University, Jinju 52828, Republic of Korea

**Keywords:** Isoprenoids, carotenoids, MVA pathway, Microbial cell factory, synthetic biology

## Abstract

Natural products, especially isoprenoids have many industrial applications, including medicine, fragrances, food additives, personal care and cosmetics, colorants, and even advanced biofuels. Recent advancements in metabolic engineering with synthetic biology and systems biology have drawn increased interest in microbial-based isoprenoid production. In order to engineer microorganisms to produce a large amount of value-added isoprenoids, great efforts have been made by employing various strategies from synthetic biology and systems biology. We also have engineered *E. coli* to produce various isoprenoids by targeting and engineering the isoprenoid biosynthetic pathways, methylerythritol phosphate (MEP), and mevalonate (MVA) pathways. Here, we introduced new combinations of the MVA pathway in *E. coli* with genes from biosafety level 1 (BSL 1) organisms. The reconstituted MVA pathway constructs (pSCS) are not only preferred to the living modified organism (LMO) regulation, but they also improved carotenoid production. In addition, the pSCS constructs resulted in enhanced lycopene production and cell-specific productivity compared to the previous MVA pathway combination (pSNA) in fed-batch fermentation. The pSCS constructs would not only bring an increase in isoprenoid production in *E. coli*, but they could be an efficient system to be applied for the industrial production of isoprenoids with industry-preferred genetic combinations.

## Introduction

Isoprenoids (well known as terpenoids) are the most abundant natural products, which contain more than 65,000 compounds with many biological functions and wide industrial applicability [1, 2]. They play a vital role in all organisms with intra- and intercellular activities, from cell integrity to energy supply: structural cholesterol and steroid hormones in mammals, photosynthetic pigments (phytol, carotenoids, etc.) in plants and ubiquinone, plastoquinone in bacteria, and mediators of polysaccharide assembly, communication and defense mechanisms [2, 3]. With the aforementioned various biological functions, they are industry-relevant chemicals: colorants, flavors, fragrances, plant hormones (agriculture), nutraceuticals, pharmaceuticals, industrial chemicals, and fuel/fuel additives [4, 5]. Despite their structural diversities, all isoprenoid biosynthesis is generated from the simple C5 building blocks, isopentenyl diphosphate (IPP) and dimethylallyl diphosphate (DMAPP). Both precursors are synthesized by two distinct biosynthetic pathways, MEP and MVA pathways. The MEP pathway, which starts from the condensation of glyceraldehyde 3-phosphate (G3P) and pyruvate, is generally present in most eubacteria, photosynthetic bacteria, and plastids in plants, while the MVA pathway, beginning with acetyl-CoA, is found in most eukaryotes, archaea, and cytosol in plants. Because microorganisms only possess either pathway, they have been engineered to utilize both pathways to improve various isoprenoid production [6]. Recent advancements in the metabolic engineering of microorganisms with synthetic biology and systems biology have resulted in successful industrial microbial cell factories (MCF) [7] and have gained increased attention in microbial-based isoprenoid production [2, 8, 9]. Microbial-based chemical production has many advantages over traditional extraction and chemical synthesis methods in the manner of environmental concerns and sustainability [10, 11]. *E. coli* is one of the representatives of prokaryotic MCF organisms and is the most tractable organism by applying metabolic engineering strategies for diverse isoprenoid products *via* their well-understood metabolic pathways and physiology [6, 12]. Therefore, we have used *E. coli* as isoprenoid producers and our previous MVA pathway construct which has significantly improved isoprenoid production in *E. coli* [13-16]. However, most genes of the pSNA originated from biosafety level 2, which requires complicated and long processes of LMO approval for biotech utilization. Due to the difficult LMO approval processes and public concerns, the use of such constructs in the biotech industry is avoided [17, 18]. Therefore, we established the newly designed MVA pathway constructs and then one of the MVA construcs was co-transformed in *E. coli* along with a carotenoid biosynthetic pathway construct to produce colorful isoprenoids lycopene or β-carotene. Additionally, we evaluated the productivity of lycopene-producing strains with the new MVA system through fed-batch fermentation.

## Materials and Methods

### Bacterial Strains and Culture Conditions

*E. coli* DH5α strain was used for gene cloning and carotenoid production. All strain information is described in Table 1. *E. coli* DH5α strain was grown in Lysogeny Broth (LB) medium (10 g of tryptone, 5 g of yeast extract, and 10 g of sodium chloride per liter) with appropriate antibiotics when necessary (ampicillin: 100 μg/ml and chloramphenicol: 50 μg/ml) at 37°C and 250 rpm for all the cloning procedures. For lycopene and β-carotene production, *E. coli* strains for seed cultures were grown in 5 ml of LB media with appropriate antibiotics at 37°C and 250 rpm and the seed culture was inoculated for the main production cultures by adjusting at the initial OD_600_ of 0.1 with 7 ml of 2YT medium (16 g of tryptone, 10 g of yeast extract, and 5 g of sodium chloride per liter) with 2%(w/v) glycerol as a carbon source and appropriate antibiotics at 30°C and 250 rpm. The lycopene and β-carotene production were carried out in test tubes for 48 h and culture broth was taken for cell growth measurement and chemical analysis every 24 h.

### Plasmids Construction

Plasmid construction was conducted using *E. coli* DH5α and all the plasmids used in this study are shown in Table 1. The nucleotide sequences of IPP isomerase from *E. coli* and *B. subtilis* showed relatively low CAI values (0.7 for *E. coli* and 0.6 for *B. subtilis*). Therefore, we performed codon optimization using Genscript’s in-house system and the process increased the CAI values of both genes by approximately 0.85 (Table S1). The top and bottom portions of MVA genes and *fni* from *B. subtilis* were introduced in the pSTV28 vector using the restriction and ligation method. Restriction enzymes (BamHI, BglII, SalI, and XhoI: NEB) and T4 DNA ligase (NEB) were used following the manufacturer's protocol. In the case of *idi* introduction in pSCS1 and pSCS2, Gibson assembly was carried out [19]. The vector fragments for the pSCS1 and pSCS2 were prepared by using the primer sets S3EE-F/S3EE-R and S3EcoE-F/S3EcoE-R, respectively. In the case of the *E. coli* *idi* insert fragment amplification, we used the primer set Ecidi-F/Ecidi-R. Additionally, codon-optimized *E. coli* *idi* linear DNA fragment for Gibson assembly was prepared by restriction enzyme treatment (BamHI and SalI, NEB). All PCR reaction was performed using Phusion High-Fidelity DNA Polymerases (NEB) according to the manufacturer's instructions. The primer sequences used in this study are listed in Table S2. *E. coli* competent cell preparation and transformation procedures were followed by the *E. coli* KCM competent cell preparation method reported in the previous paper by Eiben *et al*. [20]. The final constructs of pSCSs were prepared from antibiotic-resistant colonies after confirmation through colony PCR and sequencing.

### Quantification of Lycopene and β-Carotene Production

To determine lycopene and β-carotene contents, the culture broths after 24 h and 48 h cultivation were centrifuged at 14,000 g for 1 min, and cells were harvested by removing the supernatant. The cells were disrupted by sonication in 1 ml of acetone and incubated at 55°C for 15 min in the dark. Following centrifugation of the extract at 14,000 g for 10 min, the acetone supernatant containing lycopene or β-carotene was transferred to a clean tube.

For the lycopene and β-carotene analysis, standard solutions were prepared by dissolving 1 mg in 1 ml of acetone and 5 mg in 10 ml of acetone, respectively. Calibration curves were obtained using freshly prepared standard solutions in the range of 0.5 to 30 mg/l. The Agilent 1290 Infinity II LC system interfaced with the Agilent Ultivo triple quadrupole mass spectrometer was used for the analysis. To determine lycopene and β-carotene, both standards and samples were separated using an Agilent Infinity Lab Poroshell 120 EC-C18 column (2.1 mm × 50 mm, 1.9 μm) with isocratic methanol/MTBE (95:5 v/v) as the mobile phase. The flow rate and column temperature were set at 0.4 ml/min and 30°C, respectively. The injection volume was 3 μl, and the total LC-MS/MS run time was 7 minutes. For LC-MS/MS analysis of lycopene and β-carotene, both positive ion and negative ion APCI were evaluated. The APCI settings were optimized for lycopene to produce the negatively charged molecular ion (M¯·) at m/z 536.4. Subsequent to collision-induced dissociation (CID) in negative ion mode, the product ion at m/z 467.3 was selectively detected using multiple reaction monitoring (MRM). The MRM dwell time was set at 100 ms. The capillary voltage was optimized at 3500 V, and the corona current was 30 μA. The fragmentor was adjusted to 180 V, and nitrogen was employed as the collision gas with a collision energy of 22 V. The gas temperature was maintained at 300°C, and the vaporizer was set to 350°C. The gas flow was 4.0 L/min, and the nebulizer was set at 40 psi. The APCI parameters for β-carotene were optimized to produce the [M+H]+ ion at m/z 537.5. After subjecting it to CID in positive ion mode, the product ion at m/z 177.1 was specifically detected using MRM. The dwell time for MRM was set at 100 ms. The capillary voltage was adjusted to 4500 V, with a corona current of 4.0 μA. The fragmentor was set to 120 V, and nitrogen served as the collision gas at an energy of 20 V. The gas temperature and vaporizer temperatures were 300°C and 350°C, respectively. The gas flow was regulated at 6.0 L/min, and the nebulizer was set to 40 psi.

### Quantification of Mevalonate

The cell cultures after 24 h and 48 h cultivation were centrifuged at 13,000 g for 10 min and the supernatants were only collected. Then the samples were acidified to pH 2 with 3M HCl and incubated at 45°C for 1 h to convert MVA to MVA lactone via acid-catalyzed esterification. Samples were then saturated with anhydrous Na_2_SO_4_ and extracted with ethyl acetate spiked with 0.25% veratraldehyde (Alfa Aesar). The solvent layer was used to analyze residual MVA concentration and Agilent Technologies 7890A gas chromatograph equipped with a flame ionization detector (FID) and 19091 N-133I HP-INNOWAX column (length, 30 m; internal diameter, 0.25 mm; film thickness, 250 μm) was used for the analysis. The analytical temperature of the GC was controlled at an initial temperature of 180°C for 1 min, then ramped to 200°C gradually at 2.5°C/min and held for 8 min. The detector temperature was maintained at 250°C.

### Fed-Batch Fermentation

To prepare the seed culture, the engineered *E. coli* was cultivated in 50 ml of 2YT media (16 g/l tryptone, 10 g/l yeast extract, and 5 g/l sodium chloride) with appropriate antibiotics (ampicillin: 100 μg/ml and chloramphenicol: 50 μg/ml) and 2% glycerol at 30°C for 4 h to reach an OD_600_ of 3-5. The seed culture was then transferred into a 2.5 L bioreactor containing 1 L fermentation media including glycerol (30 g/l); potassium phosphate dibasic anhydrous (13.3 g/l); ammonium phosphate dibasic (4 g/l); magnesium sulfate heptahydrate (1.2 g/l); citric acid anhydrous (1.7 g/l); yeast extract (3 g/l); thiamine hydrochloride (0.0045 g/l); trace metal solution (3 ml/l). The trace metal solution contains EDTA (2.8 g/l), zinc acetate hydrate (4.33 g/l), manganese (II) chloride tetrahydrate (5 g/l), boric acid (1 g/l), iron (III) citrate hydrate (33.33 g/l), copper (II) chloride (0.5 g/l), cobalt (II) chloride hexahydrate (0.83 g/l), and sodium molybdate (VI) dihydrate (0.83 g/l). Fed-batch fermentation was carried out at 30°C, and pH was controlled at around 6.9. DO-stat and pH-stat were used as feeding strategies, in which 100 ml feeding solution was automatically supplied as soon as the dissolved oxygen level or pH increased depleting the carbon source. The feeding solution for fed-batch fermentation is composed of glycerol (600 g/l), magnesium sulfate heptahydrate (15 g/l), and yeast extract (60 g/l).

### Statistical Analysis

Data on carotenoid production and cell-specific productivity from this experiment were statistically analyzed. All data are presented as the mean ± standard deviation (SD) of two or three biological replicates. Statistical significance was determined using the Student’s t-test *via* Excel’s data analysis tool, with significance levels indicated as * (*p* ≤ 0.05), ** (*p* ≤ 0.01), and *** (*p* ≤ 0.001).

## Results

### Reconstitution of MVA Pathway for *E. coli* Engineering

**Fig. 1:**
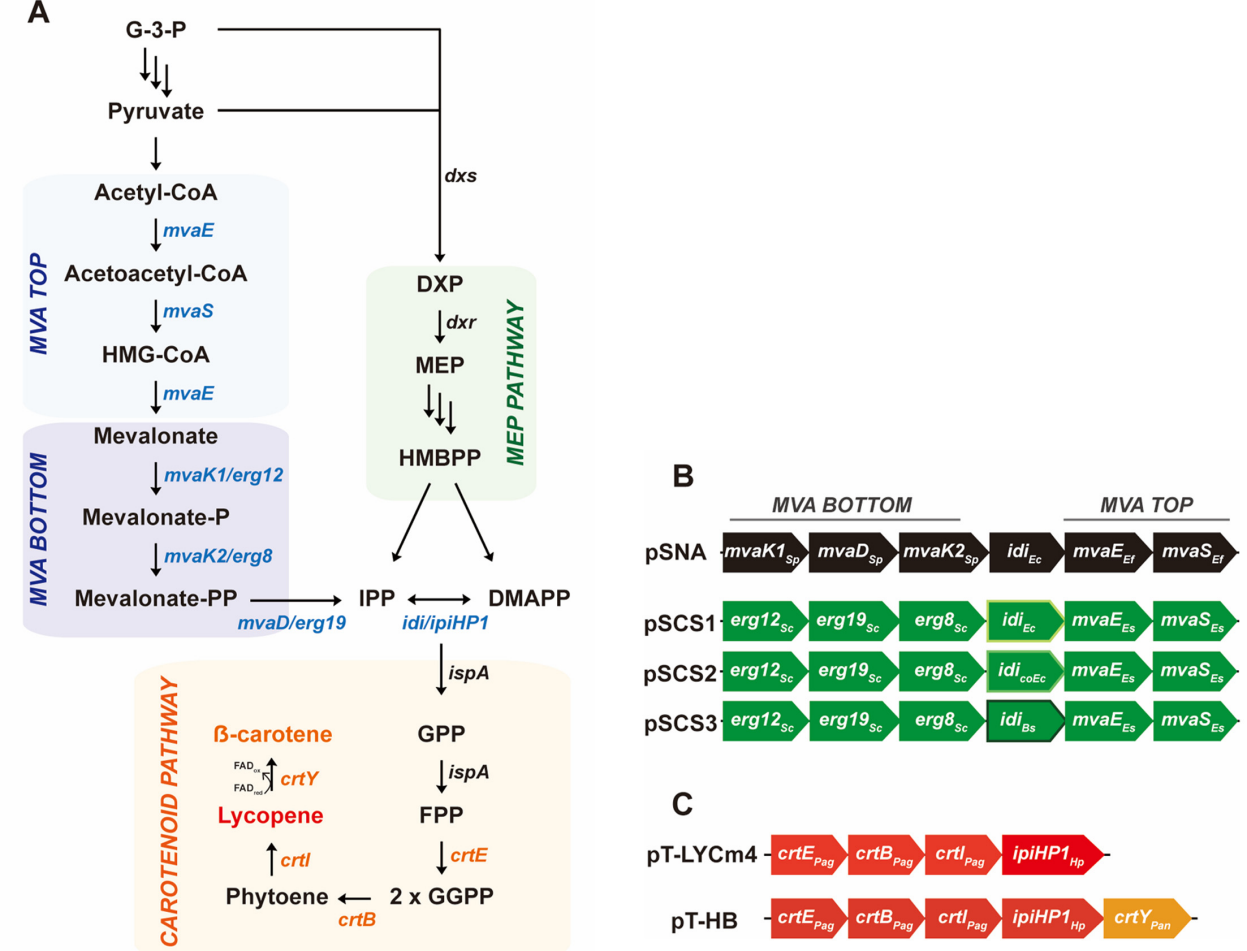
Overall experimental design of this study. (**A**) Schematic diagram of carotenoid biosynthesis in *E. coli*. The native *E. coli* MEP pathway (right) and exogenous MVA pathway (left) are shown. The biosynthesis of lycopene and β-carotene from the precursors IPP and DMAPP are described (bottom). The genes we introduced using the plasmid system are shown in blue and orange. All genes and their corresponding enzymes are the following; *mvaE*: acetoacetyl-CoA thiolase/HMG-CoA reductase, *mvaS*: HMG-CoA synthase, *mvaK1/erg12*: mevalonate kinase, *mvaK2/erg8*: phosphomevalonate kinase, *mvaD/erg19*: mevalonate 5-diphosphate decarboxylase, *Idi/ipiHP1*: IPP isomerase, *ispA*: FPP synthase, *crtE*: GGPP synthase, *crtB*: phytoene synthase, *crtI*: phytoene desaturase, *crtY*: lycopene cyclase, *dxs*: DXP synthase, *dxr*: DXP isomerase reductase. Pathway intermediates G3P: glyceraldehyde 3 phosphate, DXP: 1-deoxy-D-xylose 5 phosphate, MEP: 2-C-methyl-D-erythriol 4-phosphate, HMBPP: 1-hydroxy-2-methyl-2(**E**) butenyl 4-pyrophosphate, IPP: isopentenyl diphosphate, DMAPP dimethylallyl diphosphate, GPP: geranyl pyrophosphate, FPP: farnesyl diphosphate, GGPP geranylgeranyl diphosphate. (**B**) Design of MVA pathway constructs we used in this study. Both the pSNA and pSCS constructs were divided into 3 parts: bottom, top, and IPP isomerase. The pSNA construct consisted of the top portion (*mvaE* and *mvaS* from *E. faecalis*), the bottom portion (*mvaK1*, *mvaD*, and *mvaK2* from *S. pneumoniae*), and *E. coli* *idi*. In the case of the pSCS constructs, the top portion was from *E. saccharolyticus*, and the bottom portion was from *S. cerevisiae*. IPP isomerase was prepared in 3 different versions: native *E. coli* *idi* for pSCS1, *E. coli* codon-optimized *E. coli* *idi* for pSCS2, and *B. subtilis* *fni* for pSCS3. **C**. Plasmid constructs employed in this study to facilitate carotenoid biosynthesis. pT-LYCm4 and pT-HB are introduced in *E. coli* for lycopene and β-carotene biosynthesis. The pT-LYCm4 contains *crtE*, *crtB*, and *crtI* derived from *P. agglomerans* and ipiHP1 of *H. pluvialis*. The pT-HB was constructed by introducing *crtY* from *P. ananatis* right next to the *ipiHP1* into the pT-LYCm4 plasmid construct.

Our previous studies have reported improved isoprenoid production with the supply of isoprenoid precursors through the whole MVA pathway expression by introducing the pSNA plasmid in *E. coli* (Fig. 1A) [13-16]. The pSNA comprises six genes of the MVA pathway and *idi*: the top portion from *Enterococcus faecalis*, the bottom portion from *Streptococcus pneumoniae*, and *idi* from *E. coli* (Fig. 1B). Here, we reconstituted the whole MVA pathway by replacing the genes from BSL 1 organisms. First, *erg12*, *erg19*, and *erg8*, the bottom portion of MVA pathway genes from *Saccharomyces cerevisiae* were introduced in pSTV28. Then we performed sequence homology analysis using the BLAST program to replace the top portion genes *mvaE* and *mvaS* from *E. faecalis*. Putative protein sequences from *Enterococcus saccharolyticus* with 62.7% and 74.9% sequence similarity to *E. faecalis* *mvaE* and *mvaS*, respectively were identified and introduced in the pSTV28. Additionally, we placed three different versions of IPP isomerases (native *E. coli* *idi*, codon-optimized *E. coli* *idi*, and *B. subtilis* *fni*) between the top and bottom portion of the MVA construct and named the constructs as pSCS1, pSCS2, and pSCS3, respectively (Fig. 1B and Table 1).

### Evaluation of Reconstituted MVA Pathway for Carotenoid Biosynthesis in *E. coli*

To validate the function and efficiency of pSCS1, 2, and 3 (pSCSs) in *E. coli*, we engineered the *E. coli* strains to produce color pigment isoprenoids, lycopene or β-carotene. With the purpose of carotenoid production, carotenoid biosynthetic pathway constructs, pT-LYCm4 [21] or pT-HB [22] (Fig. 1C) was co-transformed with one of the MVA pathway constructs (pSNA, pSCS1, 2, or 3, Fig. 1B) or empty plasmid (pSTV28) as a control strain in *E. coli* (Table 1).

**Fig. 2:**
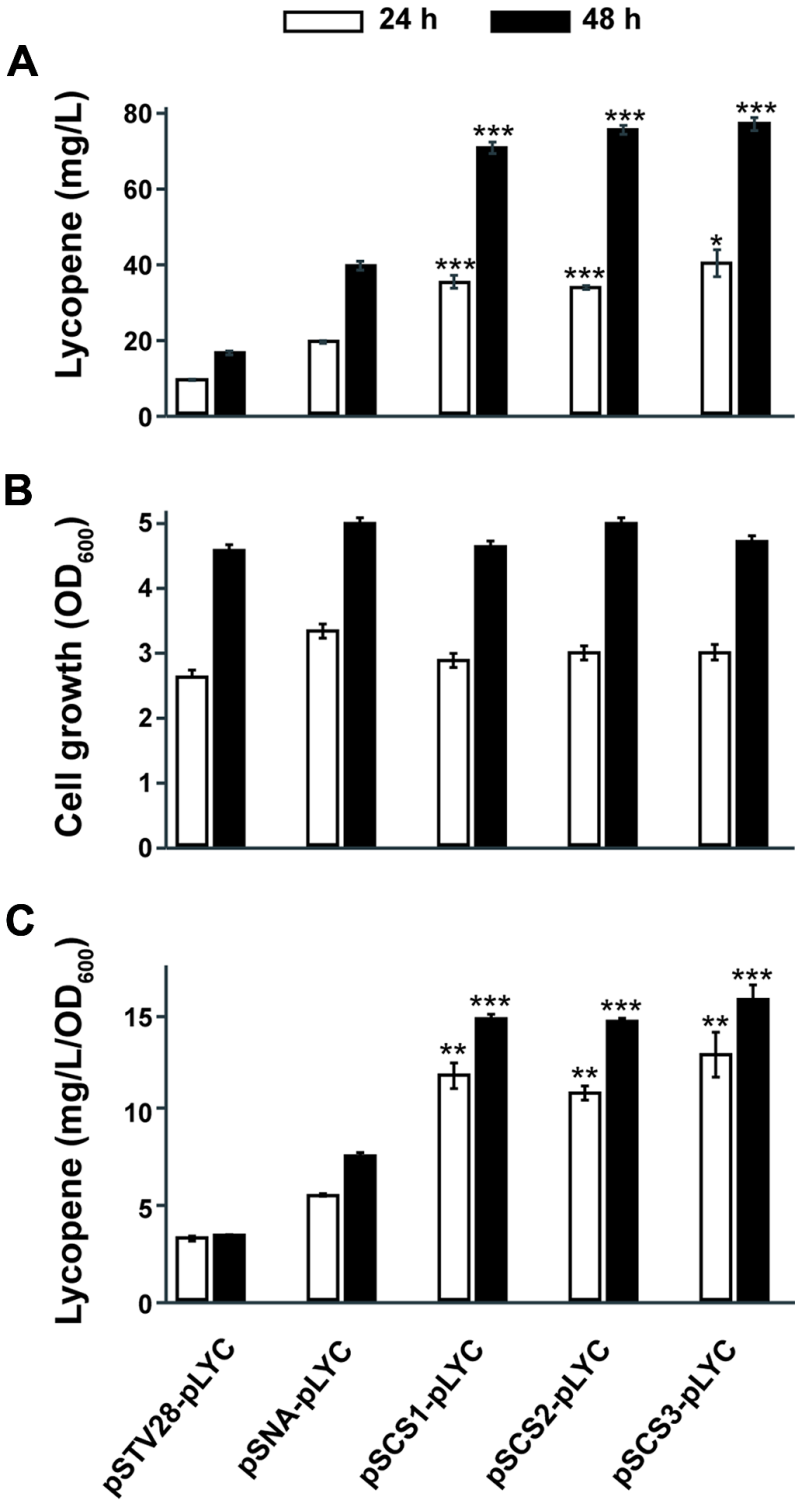
Lycopene production and cell growth in test tube culture. (**A**) Lycopene production in pSTV28-pLYC (no MVA expression), pSNA-pLYC (BSL2 MVA construct), pSCS1-pLYC, pSCS2-pLYC, and pSCS3-pLYC (BSL1 MVA constructs) after 24 hr and 48 hr cultivation (**B**) Cell growth measurement at OD_600_. (**C**) Cell-specific productivity of lycopene. Data are shown as mean ± SD of three biological replicates. Asterisks in panels A and C indicate statistical significance as follows: * (*p* ≤ 0.05), ** (*p* ≤ 0.01), and *** (*p* ≤ 0.001), when compared to the pSNA-pLYC strain.

**Lycopene Production in *E. coli***. The introduction of the MVA pathway significantly improved lycopene production without exhibiting any growth inhibition in *E. coli* (Fig. 2). The pSTV28-pLYC strain produced approximately 9 mg/l of lycopene after 24 h of cultivation, resulting in a pale red color. In contrast, strains containing MVA pathway construct, pSNA or pSCSs, produced 19-40 mg/l of lycopene, leading to a deep red color (Figs. 2A and S1). In addition, the newly designed pSCS-harboring strains exhibited a significant increase in lycopene production compared to the pSNA-containing *E. coli*. Among the strains, the pSCS3-pLYC strain reached the highest lycopene production levels at both time points, yielding 39 mg/l at 24 h and 77 mg/l at 48 h. This represents an increase of 111% and 97% compared to the pSNA-pLYC strain, which produced 19 mg/l at 24 h and 39 mg/l at 48 h, respectively (Fig. 2A and 2B). Moreover, the pSCS-containing strains resulted in around 15 mg/l/OD_600_ of cell-specific productivity, while the pSNA-pLYC reached 7.5 mg/l/OD_600_. The highest cell-specific productivity was 16 mg/l/OD_600_ in the pSCS3-pLYC at 48 h culture (Fig. 2C). The codon-optimized IPP isomerases yielded no significant improvement in either lycopene production or cell growth (Fig. 2). However, after 48 h, strains containing the codon-optimized *idi* from *E. coli* and *B. subtilis* resulted in 7% and 9% increases in lycopene production, respectively, compared to the strain harboring the native *E. coli* *idi* with statistical significance (Fig. 2). Overall, lycopene production was drastically enhanced by the expression of MVA pathway (Fig. 2A). Lycopene cell-specific productivity in the pSCS-introduced strains pSCS1-pLYC, pSCS2-pLYC, and pSCS3-pLYC, improved by 95%, 93%, and 109%, respectively, at 48 h compared to the pSNA-pLYC (Fig. 2C).

**Fig. 3:**
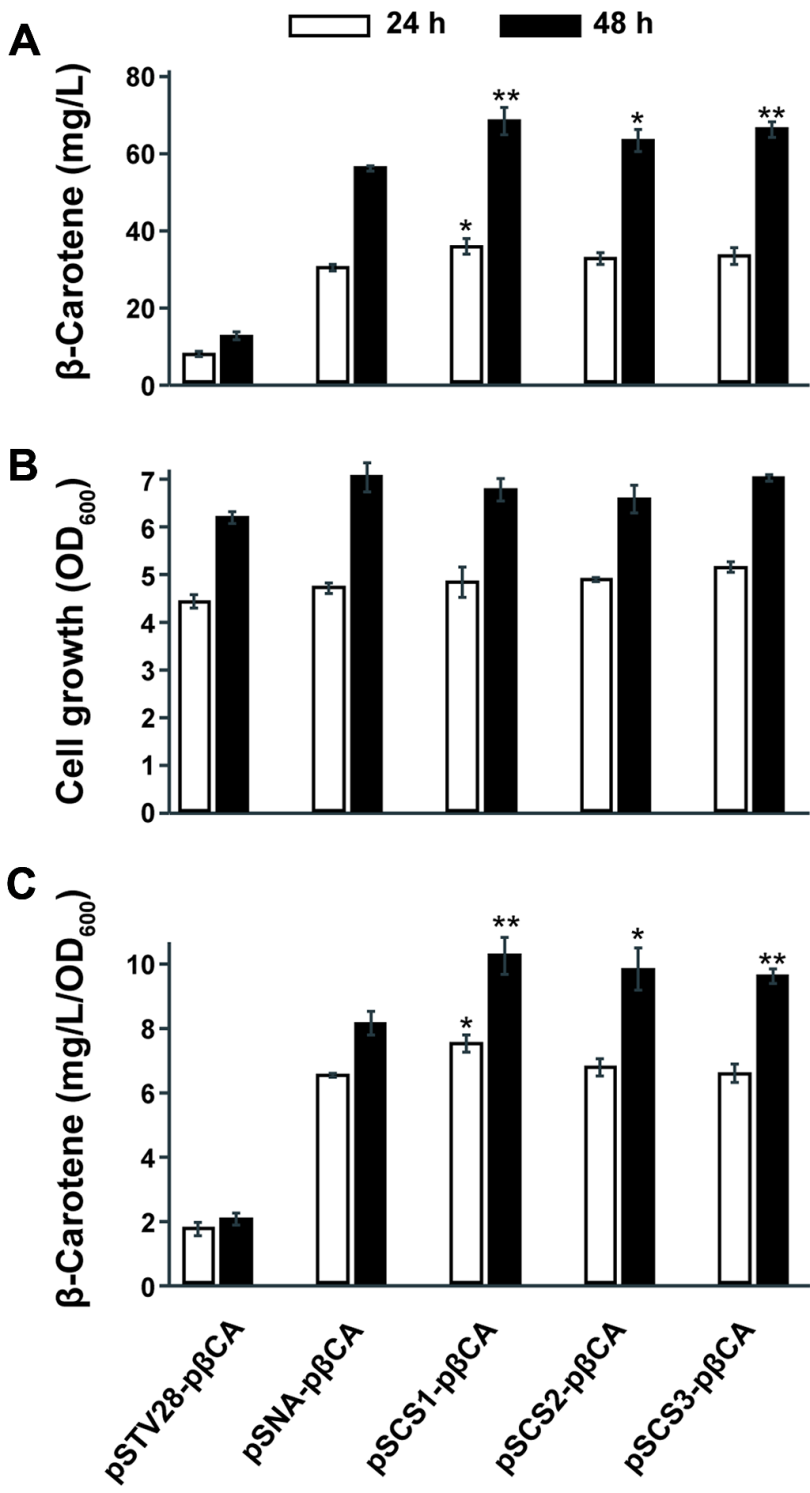
β-Carotene production and cell growth in test tube culture. (**A**) β-carotene roduction in pSTV28-pβCA (no MVA expression), pSNA-pβCA (BSL2 MVA construct), pSCS1- pβCA, pSCS2- pβCA, and pSCS3-pβCA (BSL1 MVA constructs) after 24 h and 48 h cultivation (**B**) Cell growth measurement at OD_600_. (**C**) Cell-specific productivity of β-carotene. Data are shown as average ± SD of three biological replicates. Asterisks in panels A and C indicate statistical significance as follows: * (*p* ≤ 0.05), and ** (*p* ≤ 0.01), compared to the pSNA-pβCA strain.

**Beta-Carotene Production in *E. coli*.** Similar to lycopene production, the expression of the MVA pathway greatly improved β-carotene production in *E. coli* (Fig. 3A). The native MEP pathway alone for the precursor supply seems not to be sufficient to improve the β-carotene production and no improvement in production and growth was found in the pSTV28-pβCA strain indeed (Fig. 3A and 3B). The pSTV28-pβCA produced approximately 12.5 mg/l of β-carotene at 48 h cultivation. The introduction of pSNA, pSCS1, pSCS2, or pSCS3 with pT-HB reached 57.6 mg/l, 70.1 mg/l, 65.1 mg/l, and 68 mg/l of -carotene production at 48 h, respectively, which is over 300% improvement (Fig. 3A), and deep orange colors were definite in the presence of MVA pathway expression (Fig. S1). The strains with MVA pathway expression led to an increase of β-carotene production and slightly improved growth, but no significant growth difference was observed among the MVA pathway-expressing strains during 48 h cultivation (Fig. 3A and 3B). The introduction of pSCSs was more beneficial than the pSNA for β-carotene production and cell-specific productivity, but no distinct production shift was observed among the pSCS-containing strains. Unlike the lycopene production strains, the pSCS-containing strains brought only less than 25% enhancement in the production and cell-specific productivity of β-carotene in comparison to the pSNA-pβCA strain. The introduction of pSCS1 resulted in the highest production of β-carotene by about 70 mg/l at 48 h and it is 22% higher production by comparison to the pSNA-pβCA (Fig. 3A), while pSCS2-pβCA and pSCS3-pβCA were 13% and 18% increased respectively. In addition, the cell-specific productivity was 4-5 folds boosted by the introduction of the MVA pathway, and pSCSs harboring constructs showed higher cell-specific productivity than the pSNA-pβCA. The highest productivity was found in the pSCS1-pβCA and it reached 10.3 mg/l/OD_600_ and the pSCS2-pβCA, pSCS3-pβCA, and pSNA-pβCA followed by around 9.88 mg/l/OD_600_, 9.66 mg/l/OD_600_, and 8.18 mg/l/OD_600_ of cell-specific productivity, respectively at 48 h (Fig. 3C).

**Fig. 4:**
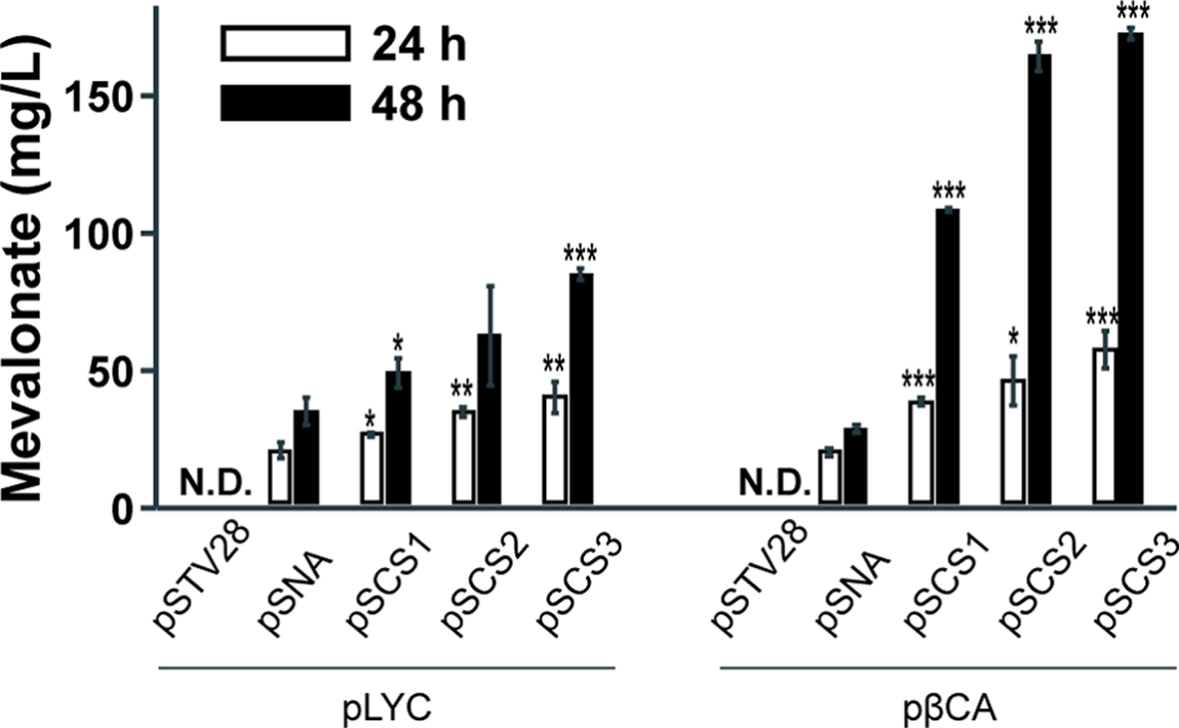
Mevalonate accumulation in lycopene and β-carotene producing *E. coli* strains at 24 h and 48 h. Data are shown as average ± SD of three biological replicates. Asterisks indicate statistical significance as follows: * (*p* ≤ 0.05), ** (*p* ≤ 0.01), and *** (*p* ≤ 0.001), when compared to the pSNA-harboring strain in each panel.

**MVA Accumulation in Engineered *E. coli*.** To evaluate the balance of the top and bottom portions of our newly reconstituted MVA pathway, we analyzed the MVA accumulation in the cell. Except for the pSTV28-containing strains (empty plasmid-no MVA pathway expression), MVA was detected in all other MVA pathway-harboring strains, and it is evidence of functional expression of *mvaE* and *mvaS* of *E. saccharolyticus* in *E. coli* (Fig. 4). In the pSNA-introducing case, about 18 mg/l and 30 mg/l of MVA were measured at 24 h and 48 h, respectively, both in lycopene and β-carotene producing-strains (Fig. 4). However, MVA accumulation was relatively higher in β-carotene producing-*E. coli* with the pSCSs. The expression of pSCS1, pSCS2, and pSCS3 resulted in 42%, 82%, and 149% of MVA enhancement in lycopene-producing strains while 296%, 505%, and 534% of more MVA were observed in β-carotene-producing constructs compared to the pSNA-harboring construct at 48 h (Fig. 4).

### Lycopene Production Profiles in Fed-Batch Fermentation

**Fig. 5:**
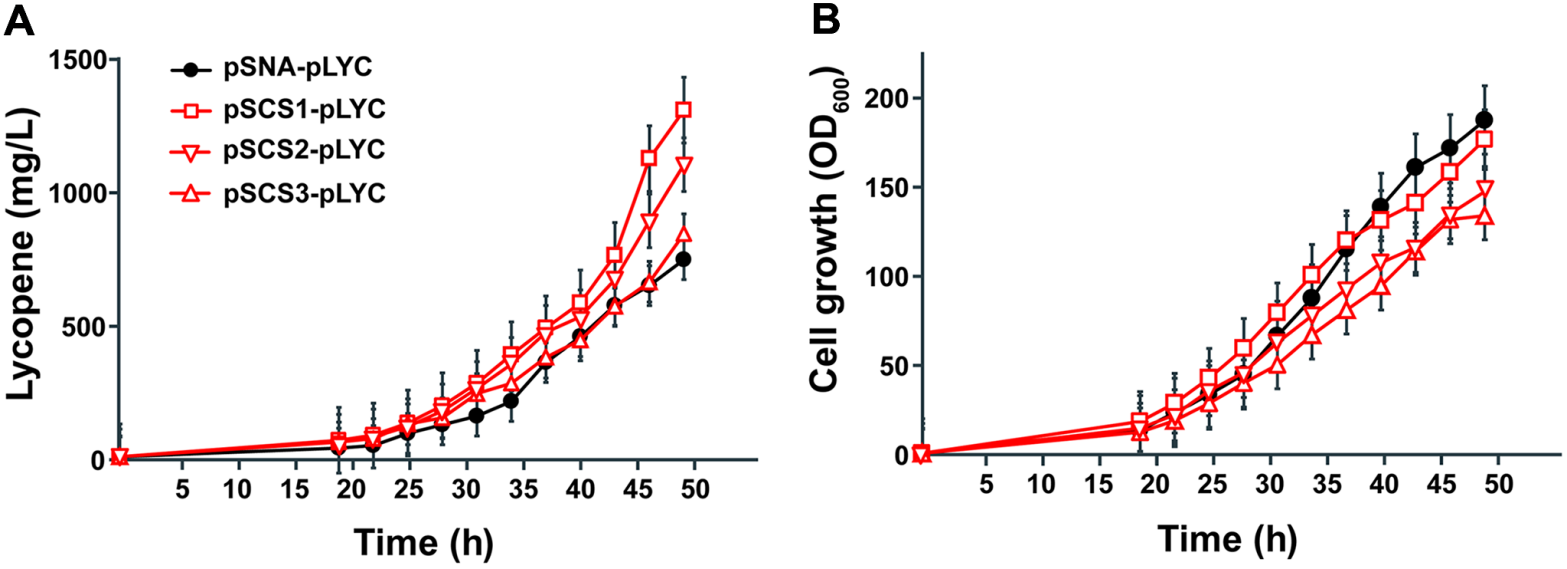
Lycopene production in 2.5 L fed-batch fermentation. (**A**) Comparison of lycopene production in pSNApLYC (circle marker), pSCS1-pLYC (square marker), pSCS2-pLYC (inverted triangle marker), and pSCS3-pLYC (triangle marker). (**B**) Cell growth (OD_600_) of the lycopene-producing *E. coli* strains. The data are shown as average ± SD of two independent replicates.

To test and compare the efficiencies of the pSCS constructs in large-scale production, we carried out fed-batch fermentation of lycopene production. The introduction of pSCSs boosted the lycopene production in fed-batch fermentation by 76% (pSCS1), 48% (pSCS2), and 13% (pSCS3) compared to the pSNA expressed strain (Fig. 5A). Among the four strains we tested, the pSCS1-pLYC strain showed the highest lycopene production at about 1.32 g/l at 49 h and the pSCS2-pLYC and pSCS3-pLYC followed the approx. 1.12 g/l and 0.85 g/l of lycopene production, respectively, while the pSNA-pLYC resulted in the lowest lycopene production (0.75 g/l) (Fig. 5A). Relatively high cell mass appeared in pSNA-pLYC and pSCS1-pLYC with the OD_600_ of 190 and 179, respectively, at the best production point. The cell-specific productivity of lycopene in pSNA-pLYC and pSCS1-pLYC reached 3.6 mg/l/OD_600_ and 7.4 mg/l/OD_600_ at 49 h (Fig. 5B). The growth in pSCS2-pLYC and pSCS3-pLYC dropped by 27% and 40% compared to the pSNA-pLYC at 49 h, but the cell-specific productivity was 2.1-fold and 1.7-fold enhanced (Fig. 5B). Even though slight changes in lycopene titer were found among the pSCS constructs in the fermentation experiment, the results consistently demonstrate around 2-fold higher lycopene production and cell-specific productivity in pSCS-containing strains like the results from test tube cultures. The glycerol consumption rates among the strains were different. The highest glycerol consumption (9.2 g/l/h) was found in the highest lycopene producer, pSCS1-pLYC strain, and the pSNA-pLYC, pSCS2-pLYC, and pSCS3-pLYC showed about 8.0 g/l/h of glycerol utilization (Fig. S1). Even though the pSNA-pLYC strain showed the best cell growth during fermentation, it reached only 3.9 mg/l/OD_600_ of cell-specific productivity, which is the lowest productivity among the strains we tested. The cell-specific productivity of pSCS1-pLYC, pSCS2-pLYC, and pSCS3-pLYC was 7.4 mg/l/OD_600_, 7.5 mg/l/OD_600_, and 6.3 mg/l/OD_600_ respectively at 49 h.

## Discussion

The introduction of an exogenous MVA pathway enables a sufficient supply of isoprenoid precursors IPP and DMAPP and it has been one of the frequent strategies to improve isoprenoid production in *E. coli* [13-16, 21-25]. Our previous studies also have reported improved isoprenoid production by expressing the whole MVA pathway using the pSNA plasmid system [13-16]. The pSNA is constituted with genes originating from BSL2 organisms, *E. faecalis* and *S. pneumoniae* and the BSL2 of gene origins sluggish industrial accessibility and utilization for the production of industry-relevant isoprenoids in MCFs because of complex LMO approval processes and public concerns [17, 18]. Therefore, the newly constituted pSCS series has been designed to possess advantages to such issues over the previous MVA pathway construct (pSNA) by replacing the genes from BSL1 organisms. In addition, MVA accumulation in the cells has been a frequent issue when the whole MVA pathway is expressed in *E. coli* because of inefficient expression of the bottom part of MVA [26, 27]. Moreover, the polycistronic MVA operon expression under a single promoter might result in an imbalance of gene expression levels by lowering the expression of genes located far from the promoter sequences [28]. To avoid such issues, we focus on balancing the expression of the MVA bottom parts with the corresponding top pathway. First, we chose one of the well-studied and validated MVA bottom pathways from *S. cerevisiae*, which brought similar β-carotene production like the MVA bottom pathway of pSNA [13] and then located the genes *erg12*, *erg19*, and *erg8* right next to the promoter. The functionality of mvaE and mvaS from *E. saccharolyticus* has not been verified, so we performed MVA analysis in our pSCSs containing *E. coli* strains (Fig. 4). The analysis validated the functionality of the MVA top pathway from *E. saccharolyticus*, like the MVA top pathway from *E. faecalis*, through MVA accumulation in our pSCSs-containing *E. coli* strains. Because the MVA bottom pathways of pSNA and pSCSs demonstrated similar performance [13], the MVA top pathway of the pSCS series seems more efficient in synthesizing MVA than the pSNA, resulting in the enhancement of MVA and carotenoid contents in *E. coli*. The IPP isomerases we used in this study have no considerable and beneficial impacts on the growth and carotenoid production in the pSCS2 and pSCS3-containing strains. For the high utilization of pSCS constructs in the biotech industry, several issues should be clarified. The MVA accumulation was high in β-carotene producing strains compared to the lycopene-producing strains, but β-carotene production improvement was not drawn well enough in the pSCSs-containing strains. The cell-specific production of β-carotene was also lower than the lycopene-producing strains in pSCSs-containing strains. We assumed that the expression of lycopene cyclase CrtY from *Pantoea ananatis* might affect the low productivity of β-carotene in *E. coli*. Since the CrtY has been known as a flavoprotein that requires a reduced FAD as a cofactor [29], the β-carotene biosynthetic pathway may deplete *E. coli* with excessive utilization of the cofactors. This might also be one possible reason for the MVA accumulation in the pSCSs-containing β-carotene-producing strains (Fig. 4). In order to develop the β-carotene-producing strains for mass production, proper cofactor management may be necessary, and this should be addressed in future work by creating an optimized environment for CrtY activity. Additionally, we may consider the culture condition optimization for the β-carotene production. The β-carotene-producing strains displayed better growth compared to the lycopene-producing strains and it might be the culture condition is good for growth but not optimal for the β-carotene production. Unfortunately, the lycopene productivity per cell growth in fed-batch fermenter culture has not reached the productivity in test-tube culture. We presume that the higher productivity observed in test tube culture, as compared to fed-batch fermentation, resulted from specific environmental condition differences, such as oxygen level, pH, and concentrations of nutrients and byproducts. Optimizing the medium by adding auxiliary carbon sources, phosphorus (P) and nitrogen (N) sources, or using appropriate buffers, has been shown to improve lycopene production [30]. Additionally, adjusting pH and oxygen levels can significantly impact cell growth and lycopene overproduction [31]. By incorporating these environmental factors into future studies, we will identify the conditions that could ultimately facilitate successful mass production of carotenoids through fed-batch fermentation.

In this study, the efficiencies of the newly designed MVA pathway, pSCS series, have been evaluated by analyzing colorful carotenoid production and MVA accumulation. Based on the results, we successfully reconstituted the MVA operon with genes from BSL1 organisms by providing suitable and applicable genetic sources for various isoprenoid production in the biotech industry, which is beneficial to intricate LMO regulation and public concerns. However, it might be necessary to discover or engineer a powerful MVA bottom pathway for the enhancement of isoprenoid production without the MVA accumulation in *E. coli*.

## Supplemental Materials

Supplementary data for this paper are available on-line only at http://jmb.or.kr.



## Figures and Tables

**Table 1:** Plasmids and strains used in this study.

Plasmids	Description	Ref.
pSTV28	P_lac_, pACYC184 ori, *lacZ*, Cm^r^	Takara Co., Ltd.
pTrc99A	P_trc_, pBR322 ori, lacI^q^, Amp^r^	Amersham Biosci.
pSNA	pSTV28 containing *mvaE* and *mvaS* of *E. faecalis*, *mvaK1*, *mvaK2*, and *mvaD* of *S. pneumoniae*, and *idi* of *E. coli*	[2]
pT-LYCm4	pTrc99A with *crtE*, *crtB*, and *crtI* of *Pantoea agglomerans*, and *ipiHP1* of *H. pluvialis*	[6]
pT-HB	pT-LYCm4 with *crtY* of *Pantoea ananatis*	[7]
pSCS1	pSTV28 containing *E. coli* codon-optimized *EsmvaE* and *EsmvaS* from *E. saccharolyticus*, *E. coli* codon-optimized *erg12*, *erg19*, and *erg8* from *S. cerevisiae* and *idi* from *E. coli*	This study
pSCS2	pSTV28 containing *E. coli* codon-optimized *EsmvaE* and *EsmvaS* from *E. saccharolyticus*, *E. coli* codon-optimized *erg12*, *erg19*, and *erg8* from *S. cerevisiae* and *E. coli* codonoptimized *idi* from *E. coli*	This study
pSCS3	pSTV28 containing *E. coli* codon-optimized *EsmvaE* and *EsmvaS* from *E. saccharolyticus*, *E. coli* codon-optimized *erg12*, *erg19*, and erg8 from *S. cerevisiae* and *E. coli* codonoptimized *fni* from *B. subtilis*	This study
Strains	Description	Ref.
DH5α	F−, ϕ80dlacZΔM15, Δ(lacZYA-argF)U169, deoR, recA1 endA1, hsdR17(rK− mK+), phoA, supE44, λ−, thi-1, gyrA96, relA1	Lab collection
pSTV28-pLYC	*E. coli* harboring pSTV28 and pT-LYCm4	This study
pSNA-pLYC	*E. coli* harboring pSNA and pT-LYCm4	This study
pSCS1-pLYC	*E. coli* harboring pSCS1 and pT-LYCm4	This study
pSCS2-pLYC	*E. coli* harboring pSCS2 and pT-LYCm4	This study
pSCS3-pLYC	*E. coli* harboring pSCS3 and pT-LYCm4	This study
pSTV28-pβCA	*E. coli* harboring pSTV28 and pT-HB	This study
pSNA-pβCA	*E. coli* harboring pSNA and pT-HB	This study
pSCS1-pβCA	*E. coli* harboring pSCS1and pT-HB	This study
pSCS2-pβCA	*E. coli* harboring pSCS2 and pT-HB	This study
pSCS3-pβCA	*E. coli* harboring pSCS3 and pT-HB	This study
