# Supplementary material for: Reconstitution of the Mevalonate Pathway for Improvement of Isoprenoid Production and Industrial Applicability in Escherichia coli
Source: J Microbiol Biotechnol. 2024 Oct 11;34(11):2338–46. doi: 10.4014/jmb.2408.08053 (PMC11637829; doi:10.4014/jmb.2408.08053)
Supplement: Supplementary file 1 [file jmb-34-11-2338-supple.pdf]

J. Microbiol. Biotechnol. 2024. 34(11): 2338–2346
https://doi.org/10.4014/jmb.2408.08053

Supplementary Figures and Tables

Reconstitution of the mevalonate pathway for improvement of isoprenoid production and industrial applicability in *Escherichia coli*

Min-Kyoung Kang, Minh Phuong Nguyen, Sang-Hwal Yoon, Keerthi B. Jayasundera, Jong-Wook Son, Chonglong Wang, Moonhyuk Kwon, and Seon-Won Kim

Figure S1. Color comparison of *E. coli* strains producing lycopene and beta-carotene in test tubes after 24 h and 48 h cultivation in 2YT medium with 2% (v/v) glycerol as a carbon source and appropriate antibiotics at 30˚C and 250 rpm.

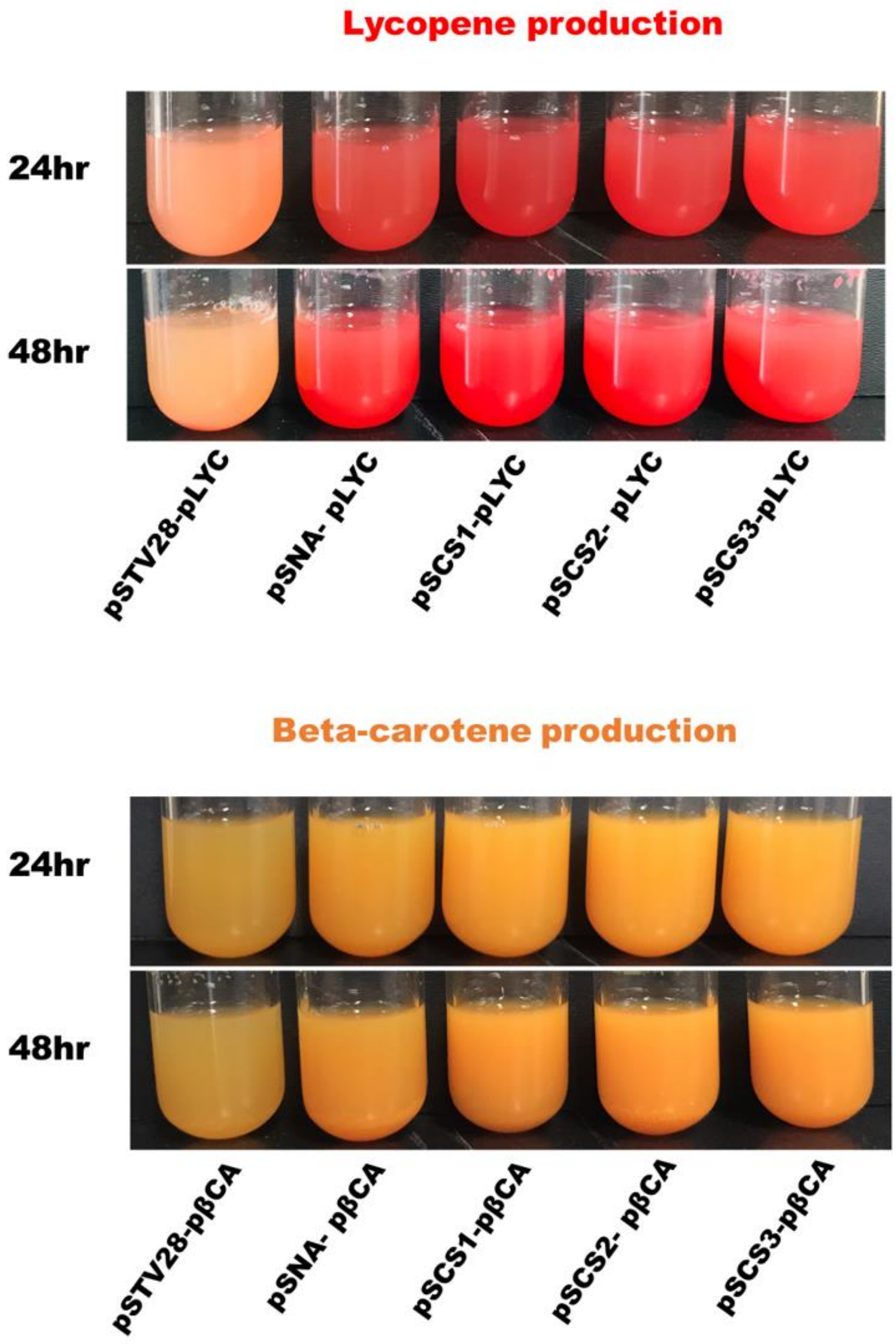

Figure S2. Profiles of fed-batch fermentation of lycopene-producing *E. coli* strains. Time-course trends of dissolved oxygen, aeration, RPM, pH, and pure oxygen supplement during fed-batch fermentation of pSNA-pLYC, pSCS1-pLYC, pSCS2-pLYC, and pSCS3-pLYC. Feeding interval and volume adjustment were also indicated. When the culture volume was over 1,500 mL by feeding, 500 mL of culture broth was removed.

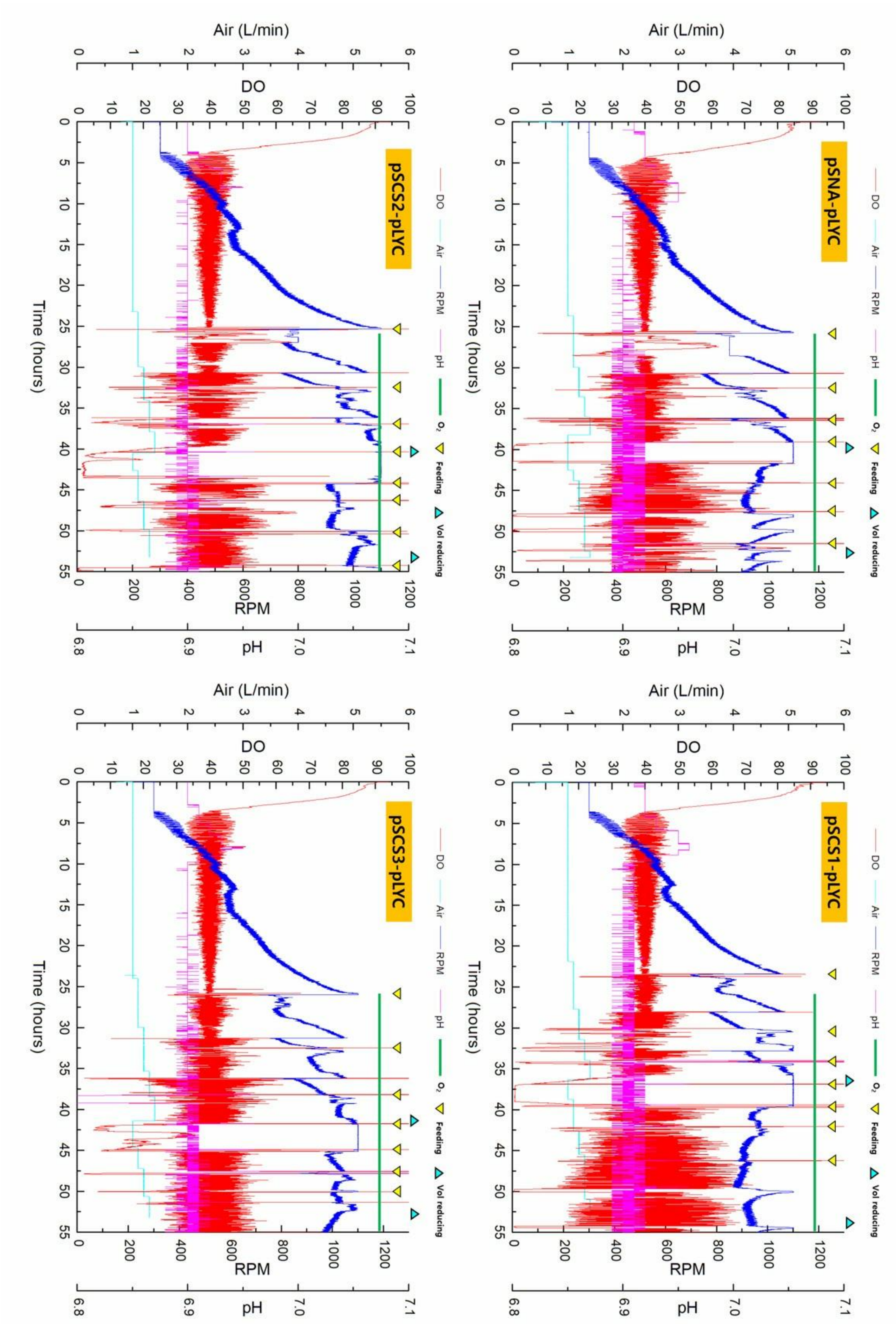

Table S1. The nucleotide sequences of codon-optimized IPP isomerases.

Gene	Sequences	CAI
coEcidi	ATGCAGACCGAACACGTGATTCTGCTGAACGCACAAGGCGT<br>TCCGACCGGTACCCTGGAAAAATATGCGGCACATACCGCAG<br>ATACCCGTCTGCATCTGGCATTTAGCAGTTGGCTGTTCAACG<br>CAAAAGGTCAGCTGCTGGTTACCCGTCGCGCACTGAGCAAA<br>AAAGCCTGGCCGGGCGTTTGGACCAATTCAGTTTGCGGTCA<br>TCCGCAACTGGGCGAATCAAACGAAGACGCGGTTATTCGTC<br>GTTGCCGTTACGAACTGGGCGTTGAAATTACCCCGCCGGAA<br>AGCATTTACCCGGATTTTCGTTATCGCGCAACCGATCCGTCA<br>GGCATTGTGGAAAACGAAGTTTGCCCGGTTTTTGCAGCACGT<br>ACCACCTCAGCACTGCAAATCAACGACGACGAAGTGATGGA<br>CTATCAGTGGTGCGATCTGGCAGACGTTCTGCACGGTATTGA<br>CGCAACCCCGTGGGCATTTAGTCCGTGGATGGTTATGCAGG<br>CAACCAATCGCGAAGCACGTAAACGTCTGAGCGCATTTACCC<br>AGCTGAAATAA	0.852
coBsfni	ATGACCCGCGCAGAACGTAAACGTCAGCATATCAACCACGC<br>GCTGAGCATTGGTCAGAAACGCGAAACCGGTCTGGACGATA<br>TCACCTTTGTGCATGTTAGTCTGCCGGATCTGGCACTGGAAC<br>AGGTGGATATCAGCACCAAAATCGGCGAACTGAGCAGCAGC<br>AGCCCGATCTTTATTAACGCAATGACCGGCGGCGGCGGTAA<br>ACTGACCTACGAAATCAACAAAAGCCTGGCACGCGCAGCAA<br>GTCAAGCAGGTATTCCGCTGGCAGTTGGTAGTCAGATGAGC<br>GCGCTGAAAGATCCGTCAGAACGTCTGAGCTACGAAATTGTG<br>CGCAAAGAGAACCCGAACGGTCTGATCTTCGCAAATCTGGG<br>TAGCGAAGCAACCGCAGCACAAGCGAAAGAAGCGGTGGAAA<br>TGATTGGCGCAAACGCACTGCAGATTCATCTGAACGTGATCC<br>AGGAAATTGTGATGCCGGAAGGCGATCGTTCTTTTAGCGGC<br>GCACTGAAACGCATTGAACAGATTTGTAGCCGCGTTTCAGTT<br>CCGGTGATCGTGAAAGAGGTGGGTTTTGGCATGAGCAAAGC<br>GAGCGCAGGTAAACTGTACGAAGCAGGCGCAGCAGCAGTTG<br>ATATTGGCGGTTACGGCGGTACCAACTTCAGCAAAATCGAGA<br>ATCTGCGTCGTCAGCGTCAGATCAGCTTTTTCAACAGTTGGG<br>GTATTAGCACCGCAGCGAGTCTGGCAGAAATTCGTAGCGAAT<br>TTCCGGCAAGTACCATGATTGCATCAGGCGGTCTGCAGGAT<br>GCACTGGACGTTGCAAAAGCGATTGCACTGGGCGCAAGTTG<br>TACCGGTATGGCAGGTCATTTCCTGAAAGCACTGACCGATAG<br>CGGCGAAGAAGGTCTGCTGGAAGAAATTCAGCTGATCCTGG<br>AGGAGCTGAAACTGATCATGACCGTTCTGGGCGCACGTACC<br>ATTGCGGATCTGCAAAAAGCACCGCTGGTGATTAAAGGCGAA<br>ACCCATCATTGGCTGACCGAACGCGGCGTTAATACCAGTAG<br>CTATAGCGTGCGTTAA	0.845

Table S2. **Primers used in this study.**

Primers	(5' → 3')	Source
Ecidi-F	Forward primer for *E. coli idi* cloning (insert)<br>GAAACCTACCTGGACAAATAAGATCCAGGAGGTAACTGT	This study
Ecidi-R	Reverse primer for *E. coli idi* cloning (insert)<br>CTGCAGGTCGACTCTAAGATCTTATTTAAGCTGGGTAAATG	This study
S3EE-F	Forward primer for *E. coli idi* cloning (vector)<br>GATCTTAGAGTCGACCTGCAGG	This study
S3EE-R	Reverse primer for *E. coli idi* cloning (vector)<br>TCTTATTTGTCCAGGTAGGTTTC	This study
S3EcoE-F	Forward primer for codon-optimized *E. coli idi* cloning (vector)<br>AGCTGAAATAAGATCTTAGAGTCGACCTGCAGGCATGCAAG	This study
S3EcoE-R	Reverse primer for codon-optimized *E. coli idi* cloning (vector)<br>CATACAGTTACCTCCTGGATCTTATTTGTCCAGGTAGGTTT	This study
